# Essentials of psychoanalytic process and change: how can we investigate the neural effects of psychodynamic psychotherapy in individualized neuro-imaging?

**DOI:** 10.3389/fnhum.2013.00355

**Published:** 2013-08-02

**Authors:** Heinz Boeker, André Richter, Holger Himmighoffen, Jutta Ernst, Laura Bohleber, Elena Hofmann, Johannes Vetter, Georg Northoff

**Affiliations:** ^1^Department for Psychiatry, Psychotherapy and Psychosomatics, Centre for Depressions, Anxiety Disorders and Psychotherapy, University Hospital of Psychiatry ZurichZurich, Switzerland; ^2^Department of Psychiatry and Psychotherapy, University Hospital Zurich, University of ZurichZurich, Switzerland; ^3^Mind, Brain Imaging and Neuroethics, Institute of Mental Health Research, University of OttawaOttawa, ON, Canada

**Keywords:** psychoanalytic process, psychodynamic psychotherapy, individualized neuro-imaging, design problem, translation problem, Interpersonal Relationship Picture Set (IRPS), Operationalized Psychodynamic Diagnosis (OPD-2)

## Abstract

The paper focuses on the essentials of psychoanalytic process and change and the question of how the neural correlates and mechanisms of psychodynamic psychotherapy can be investigated. The psychoanalytic approach aims at enabling the patient to “remember, repeat, and work through” concerning explicit memory. Moreover, the relationship between analyst and patient establishes a new affective configuration which enables a reconstruction of the implicit memory. If psychic change can be achieved it corresponds to neuronal transformation. Individualized neuro-imaging requires controlling and measuring of variables that must be defined. Two main methodological problems can be distinguished: the design problem addresses the issue of how to account for functionally related variables in an experimentally independent way. The translation problem raises the question of how to bridge the gaps between different levels of the concepts presupposed in individualized neuro-imaging (e.g., the personal level of the therapist and the client, the neural level of the brain). An overview of individualized paradigms, which have been used until now is given, including Operationalized Psychodynamic Diagnosis (OPD-2) and the Maladaptive Interpersonal Patterns Q-Start (MIPQS). The development of a new paradigm that will be used in fMRI experiments, the “Interpersonal Relationship Picture Set” (IRPS), is described. Further perspectives and limitations of this new approach concerning the design and the translation problem are discussed.

## Introduction

### Neurobiological studies of psychotherapy

The recently emerged dialog between psychoanalysis and neuroscience (Shevrin et al., 1992; Solms et al., 1998; Kandel, 1999; Beutel et al., 2003; Northoff, 2007; Northoff et al., 2007) led to several empirical hypotheses and investigations of psychodynamic concepts like defense mechanisms (Shevrin et al., 2002; Boeker et al., 2006; Northoff, 2007), self (Milrod, 2002), memories (Gabbard, 2000; Mancia, 2006; Peres et al., 2008), dreams (Solms, 1995, 2000; Andrade, 2007), empathy (Gallese et al., 2007). While these originally psychodynamic concepts are currently investigated in the neuroscientific context, the neural basis of core elements of psychoanalysis and psychodynamic psychotherapy has not been elucidated yet.

Though neurobiological changes in some single cases undergoing psychodynamic psychotherapy have been reported (Viinamäki et al., 1998; Overbeck et al., 2004; Saarinen et al., 2005; Lai et al., 2007; Lehto et al., 2008; Kessler et al., 2011a, 2012), systematic and well-controlled brain imaging studies of the neural effects of psychodynamic psychotherapy are still lacking.

In contrast to psychodynamic psychotherapy, the neural effects of other forms of psychotherapy like cognitive behavioral therapy (CBT) and interpersonal therapy (IPT) have been studied in brain imaging more often (see Roffman et al., 2005; Linden, 2006; Beauregard, 2007; Frewen et al., 2008; for reviews). These studies demonstrated neural modulation in various brain regions encompassing subcortical as well as medial and lateral cortical regions during predominantly cognitive-emotion regulation tasks before and after CBT or IPT. Interpretation of these findings is however constrained by various methodological problems; these include issues like objectification and quantification of the effects of psychotherapy in behavioral and subjective parameters, selection of the activation task in functional imaging, appropriate control groups, physiological, behavioral, and psychological variables indicating task-specific effects of neural stimulation, distinction between the target symptom and its possible underlying psychodynamic processes, etc. (see Frewen et al., 2008, for a detailed discussion).

While brain imaging studies of both CBT and IPT are already confronted with numerous methodological problems, the situation is even more difficult in the case of psychodynamic psychotherapy. For instance, the therapeutic relationship, including transference and counter-transference, plays a much more essential role in psychodynamic psychotherapy than in CBT and IPT; this makes it necessary to include the client-therapist relationship as an intervening variable in neural analysis. Another problem is the conceptualization of psychodynamic phenomena like ego, defense mechanisms, etc., and their translation into psychological variables for subsequent experimental testing in functional brain imaging. The neuropsychoanalyst who wants to study the neural effects of psychodynamic psychotherapy is thus confronted with numerous and highly complex input variables that he needs to account and control in order to make reliable and valid investigations of the output, the neural effects, possible.

### Essentials of psychoanalytic process and change

To this end, it is necessary to describe and characterize the essential and specific aspects that account for the process and change of a patient during a psychoanalysis or psychoanalytic psychotherapy. This could be the basis for the development of meaningful research designs and paradigms. The main questions in this respect are:

- What is the process and what is changed within a psychoanalysis or psychoanalytic psychotherapy?
- Which are the mechanisms, techniques, and actions that enable psychoanalytic process and change?

Patients mostly seek psychotherapy because of distress, i.e., they suffer from psychic symptoms, dysfunctional behaviors, and/or from disturbances in their psychosocial environment (interpersonal problems, in relationships, at work etc.) with the intention to reduce and resolve the distress. Often patients also aim to achieve a greater self-understanding. Others wish to be supported emotionally and personally or receive guidance and instructions from the therapist for resolving their problems.

The specific aspects concerning the therapeutic process and change in psychoanalysis and psychoanalytic psychotherapy address not only symptoms and dysfunctional behaviors. Another objective is to find out what may lie behind them. This is connected to the fundamental psychoanalytic concept that conscious, so-called “manifest” symptoms, thoughts and actions of the patient imply an unconscious “latent” meaning and motivation. Within a psychoanalytic perspective, conscious symptoms, and disturbances are assumed to be the result of mechanisms of defense and formations of compromise, which deal with multiple pre-conscious or unconscious factors. Such dynamics have a strong impact on how one thinks, feels, and behaves. Pre-conscious or unconscious factors may constitute intrapsychic conflicts or dilemmas, wishes, expectations, fantasies, or structural psychic functions (super-ego, ego and id, self- and object-representations, capacities to regulate affects, impulses, self-esteem, relationships with others etc.).

Consequently, a basic psychoanalytic approach to enable a therapeutic process is to generate and foster a patient's insight into and understanding of these pre-conscious or unconscious aspects and parts of him- or herself. This is to make conscious what had been unconscious before, which is part of what Freud (1933) wrote: “Where id was, there shall ego be.” A fundamental psychoanalytic technique during sessions is to ask patients to report about “what comes into their heads, even if they think it is unimportant, irrelevant, or non-sensical” (Strachey, 1953), which was called by Freud “free association.” Another approach is the patient's report of dreams and the associations to them.

On the psychoanalysts' part, the correspondent technical approach is a special form of listening (“evenly suspended attention”), the use of clarification and interpretation and the formulation of hypotheses on how the patient functions mentally to establish links to unconscious conflicts and aspects; something the patient cannot perceive on his/her own and/or accept as being connected with his/her conscious thinking and current-day functioning. However, against the patient's free association, the building of links with unconscious aspects and gaining insight in oneself, resistances and transference come into play—which both can build the grounds for interpretations of the analyst. The psychoanalytic approach aims at enabling the patient to “remember, repeat, and work through” (Freud, 1914) what has been experienced in the past, repressed, or internalized. Interpretation and insight may be the start of a reorganization of thoughts—the former pre-conscious may become conscious.

Psychoanalysis focuses on childhood experiences and relations with significant others (mother, father, siblings etc.). These relations had and still have an impact on a person's life. It is expressed in current relationships of the patient in the here-and-now with important persons or the analyst. Beutel (2009) summarizes effects that early childhood interpersonal experiences have on cerebral development through genetic expression and the development of neural connections.

Freud conceived the transference of the patient (so did his followers regarding the countertransference of the analyst) as obstacles to the therapy process. The adapted concepts were the start of developing the second basic approach in psychoanalysis and psychoanalytic psychotherapy: the focus is on what is happening in the therapeutic relationship on the basis of transference and countertransference. Dysfunctional, maladaptive relationship patterns, fears, and wishes of the patient tend to be repeated in the relation to the analyst. The relation toward the analyst (and the analyst itself) constitutes the groundwork for the patient's internal structure of expectations in relationships. The analytic setting fosters the evolution of these inner conceptions. Within the transference situation, unconscious processes can be actualized. Experiencing a secure attachment with the analyst the patient may be enabled to become aware and reconstruct his/her memories and relationships (that may have structured him/her) and eventually work them through.

Andrade (2005) stresses the effect of positive transference as the basis of therapeutic action. The relationship between patient and analyst promotes an identification that is based upon introjection (of a good object) and empathy and can construct a new affective organization. According to Andrade (2005), the affective nucleus fosters cognitive development. On the other hand, interpretation—as the classic method of psychoanalysis—is related to explicit memory (as part of the cortex) only and does not effectively deal with implicit memory (as part of subcortical areas). Andrade (2005) points out that implicit memory can only be seen through repetitive transference (cf. Beutel, 2009). These implicit memories are unconscious affective structures that can be emptied of their quotas of affect through transference (Andrade, 2005). A new affective configuration can be established.

The Boston Change Process Study Group (2007) depicts early childhood memories (for example, attachment patterns within the second year of life) as implicit relation knowledge. This internal configuration constitutes the intercourse with others, which becomes evident in subsequent object relations. The Boston Change Process Study Group (2007) defines the intrapsychic as interpersonal experience that is implicitly incorporated. To link therapeutic change with neuroscience Andrade (2005) deduces that “inadequate object relations can lead to neurophysiological changes and that adequate analytic relations lead to psychic changes that correspond to neuronal changes” (p. 684). As described before, introjection may be the neurochemical basis of psychic change, since new neural circuits—as a result of the secretion of neurotransmitters—develop. Also, Beutel (2009) describes the neuronal plasticity that evolves after mechanisms of learning and their repetition. In psychotherapy, these progressions take time and need affective involvement (Beutel, 2009).

Within a psychotherapeutic environment, Sterba (1934) described the “therapeutic division of the ego” into an experiencing and an observing ego. During psychoanalysis the patient's ego is at the same time remembering or working through and also analyzing this process. The conscious, reasonable, non-neurotic parts of the patient's ego can be distinguished from the unconscious, conflict-motivated, irrational portions of the ego (Sterba, 1934). This potential of the neo-cortex (analyzing subcortical activities) may be linked to neurosciences in the way Andrade (2005) explains the difference between unconscious implicit and conscious explicit memories (cf. Beauregard, 2007).

Beutel and Huber (2008) state in their review that psychoanalysis has an effect on the brain and argue that the division of psychological psychotherapy and biological psychiatry regarding a patient's treatment has become out-dated. The authors stress that today the main theorem of psychoanalysis (a major part of psychic activity remains unconscious) is a convention in neuroscience. Within psychoanalysis, patients learn to deal with their reactivation of patterns in their transference. In this sense, the reconstruction of object-related, psychic configurations resembles the therapeutic effect Andrade (2005) explained.

From a neuroscientific perspective, the problem of showing these effects within methodical borders remains a challenge for the future. For instance, Zwiebel (2007) depicts the difficultness to fully understand the functioning of the analyst. Analysts oscillate between so-called personal and technical poles when treating their patients (Zwiebel, 2007). Thus, the therapeutic process can be understood to be on a micro-psychological level that can hardly be quantified (cf. Beutel and Huber, 2008).

## Two methodological problems

How can the complexity of input variables be dealt with in order to enable future brain imaging studies in psychodynamic psychotherapy? The general aim of this paper is to discuss the variables that need to be controlled and measured in studying the neural effects of psychodynamic psychotherapy. We will discuss the various variables and the methodological problems which can be subsumed under two main headings, the design problem and the translation problem. The design problem addresses the issue of how to account for functionally related variables in an experimentally independent way. For instance, the activation tasks employed in brain imaging should somehow mirror and simulate those functional processes that are assumed to mediate the therapeutic effects of psychodynamic psychotherapy. Experimentally however, we need to measure and account for both variables in an independent way without any confusion between them. The translation problem raises the question of how to bridge the gaps between the different levels of the concepts presupposed in such investigation. The gap between the personal level of the therapist and the client on the one hand, and the neural level of the brain on the other, needs to be bridged. There is also a gap between the behavioral effects of psychodynamic psychotherapy the therapist can observe and the subjective effects the client himself experiences. Finally, the gap between the psychodynamic level of the psychodynamic psychotherapy, the psychological level of the activation task in brain imaging and the neural level of the parameters to be measured needs to be bridged. The development of bridges for the various gaps is crucial in developing an experimentally sound design that allows for valid and reliable measurement and interpretation of the data. We will discuss both problems here, the design problem and the translation problem in their various facets which will be illustrated by a specific example, the example of introjection (see below for exact definition).

### Design problem

**Figure 1:**
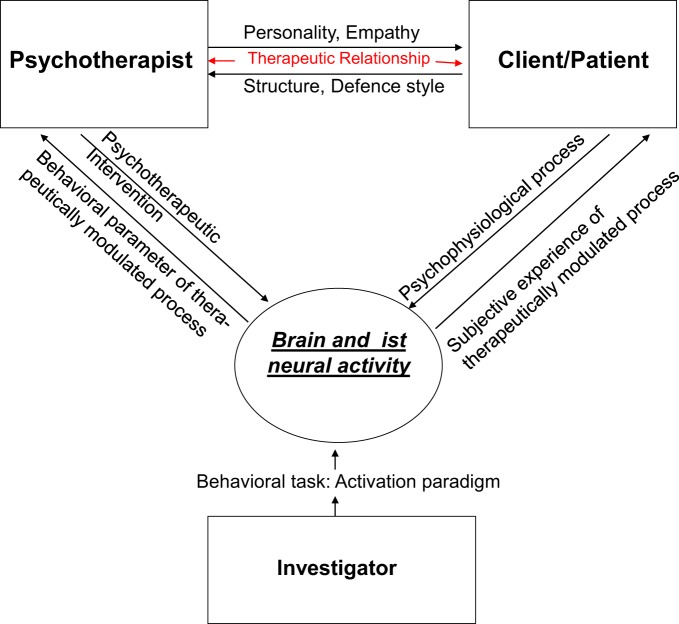
**The design problem**.

The design problem deals with the issue how to account for functionally related variables in an experimentally independent way. Relevant inputs that enter such study designs include the psychotherapist, the client, the therapeutic relationship and the investigator, i.e., the experimentator (See Figure 1 and Table 1). This discussion of the relevant inputs will shed some light on which and how their variables can be controlled and accounted for in experimental design.

**Table 1:** **Input, empirical variables, and experimental measures**.

**Input**	**Empirical variables**	**Experimental measures**
Psychotherapist	Personality, empathy	Scales for personality and empathy
	Psychotherapeutic intervention as input	Psychotherapeutic identity
	Psychotherapeutic output	Psychodynamic, subjective, and behavioral measures
	Psychodynamic process mediating between psycho-therapeutic input and output	Measurement of psychodynamic process with STIPO, OPD, etc.
	Attachment style	Adult Attachment Interview (AAI), Adult Attachment Prototype Rating (AAPR), etc.
Client/Patient	Personality and psychodynamic structure as input	Measurement of psychodynamic process with STIPO, OPD, etc.
	Behavioral and subjective input in the gestalt of symptoms	Likert scales, reaction times, and other behavioral parameters
	Therapeutically-induced changes in subjective and behavioral output	Psychophysiological measures like skin conductance, etc.
	Attachment style	Adult Attachment Interview (AAI), Adult Attachment Prototype Rating (AAPR), etc.
Patient-psychotherapist-match	Quality of therapeutic relationship	Scales for measurement of fit of match between client and therapist and thus of therapeutic relationship with Helping Alliance Questionnaire (HAQ), Vanderbilt Psychotherapy Process Scale, Working Alliance Inventory
Investigator	Concept and hypothesis of brain function	Localization vs. integration
	Behavioral task as activation paradigm and input	Neurophysiological, methodolo-gical, psychodynamic, symptom-matic, and experiential demands
	Changes in neuronal activity as output	Method of measurement (fMRI, PET, etc.)

#### The psychotherapist as “input”

What does the psychotherapist put into psychodynamic psychotherapy? First and foremost he puts in his own personality, his cognitions, his affects, and ultimately his own life history. In the further course of the interaction between the patient and the therapist it is the psychotherapist's perspective on the patient's thoughts, feelings, and behavior which essentially contributes to the development of the therapeutic relationship. Recent research demonstrated that the psychotherapist himself, as a personality with all his/her affects, cannot remain abstinent in psychotherapy as originally envisioned by Freud. If the patient experiences the analyst as an “impenetrable object,” it can lead to serious difficulties in the analytic process, e.g., the patient will transfer his/her projections onto the therapist, which in turn can trigger unconscious “hardening” by the therapist (Skogstad, 2013). Kohut (1959) pointed out that the capacity to show empathy is a major factor in how the relationship between therapist and client can develop which in turn has a strong impact on potential therapeutic effects. A recent study investigated cognitive and emotional aspects of empathy in psychotherapists (Hassenstab et al., 2007). When compared to control subjects, psychotherapists showed higher empathy scores when making inferences based on language mirroring cognitive aspects. Affective aspects of empathy did not differ between both groups though psychotherapists showed better emotion regulation with less personal distress in response to the distress of others. Though preliminary because of the low number of cases (*n* = 19), this psychological study supports the crucial importance of empathy in psychotherapists. Certainly though further studies are necessary to reveal the exact role of empathy and its distinct aspects (sensory, cognitive, affective; see also Zanocco et al., 2006) in psychotherapeutic interaction. Furthermore, one may investigate whether the neural network implicated in empathy (insula, anterior cingulate, thalamus, temporoparietal junction, amygdala; see Frewen et al., 2008) may show a higher neuronal reactivity in psychotherapists when compared to non-psychotherapists. Ideally, one would include neuronal and psychophysiological (skin conductance, heart rate, etc., see Marci and Riess, 2005) measures of the psychotherapist's emphatic abilities as confounding variables, i.e., as regressor or co-variate, in the measurement and analyses of the client's neuronal changes during psychodynamic psychotherapy.

Why consider the personality and empathic abilities of the psychotherapist as a confounding variable? Imagine, for instance, a psychotherapist with strong tendencies to identify with the patient. This, of course, enables the therapist to understand the patient and also, a client who has great difficulty internalizing significant others might well benefit from such an empathic psychotherapist and a supportive approach. On the other hand, however, it might hinder his empathic abilities and may also be problematic when he encounters for instance a depressed client who has internalized highly ambivalent object relationships. This case might be problematic for therapeutic interaction (e.g., when the transference is directed by these ambivalent aspects of the patient's internalized relationships). Sandler et al. (2011) also points out that for a successful psychotherapy beyond the actual transference relationship, which enables the transference neurosis; a different form of relationship—a “working alliance”—is required. This should also enable the patient to maintain an analytic attitude even if the transference conflicts are intense. Psychotherapeutic effects might thus not only depend on the personality and psychic structures of the psychotherapist himself but also on the specific constellation between therapist and client including their respective attachment styles (see for instance Schauenburg et al., 2006). This makes it clear that experimentally we do not only need to include personality scales for both the client and the therapist but measures for attachment styles on both sides, e.g., the Adult Attachment Interview (AAI; Hesse, 1999) or the Adult Attachment Prototype Rating (AAPR; Strauß et al., 1999).

Another variable the psychotherapist himself puts in are of course the psychotherapeutic interventions he uses to induce psychotherapeutic change; the factor accounting for the psychotherapeutic intervention may be conceptualized as “psychotherapeutic input” which describes the intervention the psychotherapist uses to induce therapeutic change in the client. Freud (1937) tackles the desired changes in psychotherapy and appropriate therapeutic interventions, when he raises the question of the “natural end of the analysis.” He emphasizes that therapeutic interventions should be aimed at overcoming the patient's inner resistances, and thus the symptoms he is suffering from will disappear. It is a question of undoing “ego-changes,” which are created by mobilizing ego-defenses against dangerously experienced drive-derivative in the course of development. This makes up the analytical process. The therapist may, for instance, maintain a state of analytic abstinence together with an evenly suspended attention as a basis for interpreting unconscious conflicts, the transference or dreams. Or he may choose to focus on working with imagination letting the client imagine various kinds of scenarios to put traumatic events into a broader context. Contrary to long-term psychoanalytical psychotherapy, the therapist may focus—within the framework of short-term psychodynamic psychotherapy—on so-called core conflictual relationship themes (CCRT) or interpersonal conflicts in the actual relationship of the patient (Luborsky et al., 1985; Luborsky and Crits-Christoph, 1989; Roth and Fonagy, 1996). This must be accounted for in a quantified and objective way as for instance by the recently developed questionnaire of psychotherapeutic identity that asks for various issues of the psychotherapists' education, experience, style, and values (see Klug et al., 2004).

In addition to psychotherapeutic input and psychodynamic process, we also need to account for the psychotherapeutic output, the effects. There have been various studies showing the therapeutic efficacy of psychodynamic psychotherapy (see for instance Leichsenring and Leibing, 2007; Haase et al., 2008; Leichsenring and Rabung, 2008; Taylor, 2008). Recently developed instruments like the Operationalized Psychodynamic Diagnosis (OPD-2; Cierpka et al., 2007; Boeker and Richter, 2008; Boeker et al., 2008; OPD-Task Force, 2008) enable an operationalized psychodynamic diagnostic approach based on a multiaxial system (consisting of four psychodynamic axes and one descriptive axis). Furthermore, OPD enables the definition of relevant therapeutic foci and the measurement of therapeutic changes (Rudolf et al., 2004). The Structured Interview for Personality Organization (STIPO; Clarkin et al., 2004) was developed according to the psychodynamic concept of Kernberg (1996). The STIPO allows the evaluation of an individual's personality organization with respect to the following dimensions: identity consolidation, quality of object relations, use of primitive defenses, quality of aggression, adaptive coping vs. character rigidity, and moral values. The psychotherapeutic output is accounted here only on a psychodynamic level; this is problematic because the measure that measures something, the psychotherapeutic output, should be different from what it shall measure, the independent variable in the experimental design (which though remains constitutively dependent on it). Therefore, what is needed additionally are some dependent variables of psychotherapeutic change and their underlying psychodynamic processes on a different level, the subjective and behavioral level.

One might argue that the neural effects themselves may well-serve as dependent though different measure of psychotherapeutic outcome. This however is to confuse different evidences. The neural effects are supposed to evidence the effects of psychotherapy on the neural level while they are not supposed to reflect evidence of the psychotherapeutic effects by themselves. We cannot measure and evidence psychotherapeutic effects by neural measures, that are supposed to mediate them if we want to avoid circularity. Hence, to reliably link neural effects to psychotherapeutic effects, we need a measure of psychotherapeutic effects that is neither psychodynamic, thereby avoiding identity with the output, nor neural in order to avoid identity with the process that is supposed to mediate its effects. As such a measure Beutel (2009) suggests changes in the known memory systems—declarative (explicit) and procedural (implicit)—, that (memory) in turn can be localized in specific brain structures. He discussed that the repression which has been overcome by analytic interventions, can lead to the repressed being recalled and then being reproduced and detected by memory tests. The findings of Nader et al. (2000) confirm the well-known fact in memory research that memory performance is affected by the constellations of encoding and retrieval situations and may distort the memory of content (“false memory,” Loftus and Ketcham, 1994). In contrast to these findings, we assume that the influence of the memory is insignificant in the constellation of the encoding and the retrieval situation, because it retrieves meaningful biographical information. Thus, memory systems can on the one hand reflect the effects of psychotherapy; on the other hand they can be localized in specific areas of the brain itself. However, this requires a careful conceptualization of such experiments: first, the confounding variables should be detected (e.g., influencing memory performance by the current emotional state of the patient/subject) and controlled, and secondly a careful selection of test instruments should be made. Only then can the memory performance be a measure that maps evidence of the effects of psychotherapy on the one hand, and locates and maps the neural level on the other hand.

#### The client as “input”

First and foremost, the client comes with a specific psychodynamic constellation and his particular personality, his psychodynamic and personality input. For instance, a certain mechanism may predominate to such a degree that it becomes pathological [e.g., introjection in the “introjective type” of depressed patients (see Blatt, 1974; Boeker et al., 2000; Taylor and Richardson, 2005)]. Consequently, more mature mechanisms cannot be used. The psychodynamic constellation of the client needs to be objectified and verified and several instruments like the OPD-2, the STIPO, and the KAPP (Weinryb et al., 1991a,b) have been developed for this purpose. These three are rating instruments based on psychoanalytical theory to assess relatively stable modes of mental functioning as they appear in self-perception of the own personality and interpersonal relations. In addition, one should also include measures of the personality like the Temperament Character Inventory (TCI) that measures various dimensions of reward (reward dependence, novelty seeking, etc.) and self (self-directedness, self-transcendence, etc.).

One possible confounding factor in experimental neuro-imaging studies of patients in psychodynamic psychotherapy could be a potentially conflicting situation of the “patients” being at the same time the “subjects” in the neuro-imaging study, as their willingness to participate in the study can be seen under the point of view of their transference situation.

However, the client does not come to the psychotherapist because of his specific psychodynamic constellation. He comes because he encounters some behavioral and subjective problems which outside observers may call symptoms. These symptoms are the aim and targets of the psychotherapeutic intervention. For instance, a client with high degree of introjection does not come because of his abnormally high introjection but because he may be severely depressed and it is his depressive symptoms that are the target of psychotherapeutic intervention. What we need to account for experimentally is thus the behavioral and subjective problems encountered by the client, i.e., his symptoms. They may for instance be measured subjectively with scales like the Beck Depression Inventory (BDI; Beck et al., 1996) or the Beck Hopelessness Scale (BHS) where the client himself rates and evaluates his subjective and behavioral problems. Or the client's problems may be rated objectively by somebody else using for instance the Hamilton Depression Rating scale (HDRS). In order to avoid confusion between psychotherapeutic intervention and symptom measurement, objective scales shall be accounted by a person that is different and independent of the psychotherapist himself since otherwise some bias and contamination by the latter cannot be excluded. Most importantly, what is needed here in the future is a clear empirical linkage between specific psychodynamic processes and particular symptoms, i.e., behavioral and subjective abnormalities. For instance, introjection or anaclitic needs have often been associated with depression (see Blatt, 1974). Referring to the psychotherapeutic context of introjection, Blatt's distinction between introjective and anaclitic depression is of special importance. Patients suffering from anaclitic depression are primarily preoccupied with issues of interpersonal relatedness (e.g., trust, caring, intimacy, and sexuality) and use primarily avoidant defenses (e.g., denial and repression) to cope with psychological conflict and stress. In contrast, patients suffering from introjective depression are primarily concerned with establishing and maintaining a viable sense of self, ranging from a basic sense of despair, to concerns about autonomy and control, to issues of self-worth, and use primarily counteractive defenses (e.g., projection, doing and undoing, intellectualization, reaction formation, and over-compensation). Interestingly, this differentiation is significantly related to different kinds of outcome in long-term intensive treatment of seriously disturbed young adults, and different responses to two forms of therapy—psychoanalysis and psychotherapy (cf. Blatt, 1993). What is needed are studies to show the correlation between both psychodynamic and symptomatic measures entailing what we call psychodynamic-symptomatic specificity.

Finally, we need to account for the change in the client as induced by the psychotherapy. These changes may be measured in behavioral and psychodynamic terms as discussed above and should also be accounted for in subjective terms. For instance, one hypothesis is that introjection may be accounted for by what we call self-related processing (Boeker and Richter, 2008; Northoff, 2008). If so one would expect increased self-relatedness in depressed patients when compared to healthy subjects which is indeed the case as demonstrated recently (Northoff, 2007; Grimm et al., 2009). Psychodynamic psychotherapy should lead to a decrease in the self-focus in depressed patients which ideally should be accompanied by decreased introjection. If so, the subjective experience of self-relatedness may be taken as a marker of subjective change induced by psychodynamic psychotherapy. This may be accompanied ideally by behavioral markers like reaction time measures during tasks implicating self-relatedness. Most importantly, the subjective and behavioral measure of self-relatedness should be sensitive to both, the psychodynamic processes as induced by psychotherapeutic intervention, and the symptoms, i.e., the clients' behavioral and subjective input. This means that self-relatedness should serve as dependent variable of both introjection and depressed symptoms and that the latter two should also be linked in functional regard. All three, self-relatedness, introjection, and depressed symptoms are thus closely linked to each other in functional and hence constitutional regard while experimentally they should be kept distinct and separate. We are thus again confronted with the discrepancy between clinical and experimental levels encountering the constitution of clinical symptoms by various interdependent functions which though experimentally need to be kept apart and thus independent of each other.

#### The therapeutic relationship as input

Over the past decades the psychoanalytical situation was re-conceptualized as a dyadic system in which the psychoanalytic psychotherapist is both participant and observer. The broadened definition of counter-transference and the influence of object relations theory and various intersubjective perspectives have led to increased emphasis on the relationship between psychotherapist and patient. Many new terms have been coined to emphasize various facets of the “two-personness” of analysis including the therapeutic alliance and the “real” relationship (cf. Vaughan and Roose, 2000). The most far-reaching attempt to distinguish transference-countertransference from “reality” aspects of the dyad has occurred in the context of the growing emphasis on patient-therapist match.

Kantrowitz et al. (1989) defined match in the following way: match is “a broader field of phenomena in which counter-transference is included as one of many types of match. The individual history, characteristics, attitudes, and values of each analyst and patient predispose them respectively to certain counter-transference and transference reactions. Match, however, can also refer to observable styles, attitudes, and personal characteristics which are rooted in residual and unanalyzed conflicts, shared or triggered in any patient-analyst pair” (Kantrowitz et al., 1989, p. 895). Different types of facilitating and impeding matches are distinguished from one another which based on similarity and complementarity very much resemble the concordant and complementary transference-countertransference paradigms delineated by Racker (1968) within an object relations model. The importance of interactive, non-verbal affective communication that shapes the behavior and response of the patient and the therapist also needs to be pointed out as one central factor constituting the match (cf. Kantrowitz, 1995).

Some psychotherapy studies have focused on the question of what constitutes a good match. Luborsky et al. (1988) observed that from ten pre-treatment demographic variables (age, marital status, having children, religion and level of religious activity, education, cognitive style, etc.) only match in marital status was found to be significantly predictive of positive outcome (see Garfield and Bergin, 1978; Gruenbaum, 1983; Hollander-Goldfein et al., 1989, for other studies in this direction).

Recently, instruments to measure the fit or match between therapist and client have been developed. The Helping Alliance Questionnaire (Luborsky, 1984) investigates the subjective evaluation of the therapeutic relationship from the perspective of both the client and the therapist so that the correlation between both may reflect the fit or “match.” Another instrument is the Vanderbilt Psychotherapy Process Scale (O'Malley et al., 1983) that allows an evaluation of the client-therapist relationship by means of an external observer as for instance a video recording. It includes dimensions like patient involvement, therapist-offered relationship and exploratory process. Finally, the Working Alliance Inventory (Horvath and Greenberg, 1989; see also Bordin, 1975, 1976) includes 36 items to the dimensions goal, task and bond that can be evaluated by the client, the therapist, and an external observer.

Taken together, there is still a need for psychotherapeutic research that collects data from both participants in dyadic situations. To date there are only very few studies attempting to operationalize different factors of the therapeutic relationship and developing adequate paradigms using neuro-imaging approaches (see Kaechele and Buchheim, 2008).

#### The investigator as input

The investigator targets the brain; more specifically he aims to reveal the neural effects of psychodynamic psychotherapy. By developing his hypothesis about possible neural effects, he must presuppose (either implicitly or explicitly) a specific concept and theory of brain function. For instance, presupposing strict localizationism and modularity, he may hypothesize that neural activity in a specific region like the often observed abnormality in the subgenual anterior cingulate cortex (Mayberg, 2003) may be changed and normalized by psychodynamic psychotherapeutic intervention in depression. This hypothesis is based upon similar observations in CBT and pharmacotherapy (see Goldapple et al., 2004; Kennedy et al., 2007). However, these and almost any other brain imaging study on the neural effects of psychotherapy do show a wide variety of different regions showing neural changes. This puts the presupposition of strict localization into doubt and may make a different concept and theory of brain function.

Alternatively to localizationism, one may assume neuronal integration. Neuronal integration describes the coordination and adjustment of neuronal activity across multiple brain regions. The interaction between distant and remote brain areas is considered necessary for a complex function to occur, such as emotion or cognition (Price and Friston, 2002; Friston, 2003). Neuronal integration focusing on the interaction between two or more brain regions must be distinguished from neuronal segregation (Price and Friston, 2002; Friston, 2003). Here a particular cognitive or emotional function or processing capacity is ascribed to neural activity in a single area that is both necessary and sufficient; one can subsequently speak of neuronal specialization and localization. We assume that for instance mechanisms as complex emotional-cognitive interactions cannot be localized in specialized or segregated brain regions. Instead, we consider specific psychodynamic mechanisms to require interaction between different brain regions and thus neuronal integration

For neuronal integration to be possible, distant and remote brain regions have to be linked together which is provided by connectivity. Connectivity describes the relation between neural activity in different brain areas. There is anatomical connectivity for which we will use the term connections in order to clearly distinguish it from functional connectivity. In addition, Friston and Price (2001) distinguish between functional and effective connectivity: functional connectivity describes the “correlation between remote neurophysiological events” which might be due to either direct interaction between the events or other factors mediating both events. A correlation can either indicate a direct influence of one brain area on another or their indirect linkage via other factors. In the first case the correlation is due to the interaction itself whereas in the second the correlation might be due to other rather indirect factors like for example stimuli based on common inputs. In contrast, effective connectivity describes the direct interaction between brain areas, it “refers explicitly to the (direct) influence that one neural system exerts over another, either at a synaptic or population level” (Friston and Price, 2001). Here, effective connectivity is considered on the population level because this corresponds best to the level of different brain regions investigated here. For example, the prefrontal cortex might modulate its effective connectivity with subcortical regions thereby influencing specific functions like interoceptive processing.

Based upon connectivity, neural activity between distant and remote brain regions has to be adjusted, coordinated, and harmonized. Coordination and adjustment of neural activity might not be arbitrarily but guided by certain principles of neuronal integration (Northoff et al., 2004). These principles describe functional mechanisms according to which the neural activity between remote and distant brain regions is organized and coordinated. Such principles of neuronal integration might for instance include reciprocal modulation, modulation by functional unity, top-down modulation, and modulation by reversal (see Boeker et al., 2006; Northoff, 2008, for details). As hypothesized by us, each of these principles may be associated with a specific psychodynamic mechanism.

One may want to argue that the debate about the presupposed theory and concept of brain function is of mere theoretical interest while remaining empirically irrelevant. This however is to neglect that the experimental measure of neural change strongly depend upon the concepts we as investigator put into the investigation itself. If we, for instance, hypothesize a single or specific regions to be effected by psychodynamic psychotherapy, we only measure and analyze our data with regard to such localizationism. While we neither measure nor analyze brain function in orientation on for instance the above mentioned principles of neuronal integration that require different methods of analysis. This may be necessary in depression where the specific abnormality may not consist in one particular region but an abnormal reciprocal modulation between medial and lateral prefrontal cortex with both regions no longer activating in a converse, i.e., opposite and reciprocal way (see Northoff et al., 2004; Boeker et al., 2006; Grimm et al., 2006). Hence, by concentrating on changes in single regions, we may miss neural changes that are induced by psychotherapeutic effects like the normalization of for instance reciprocal modulation between medial and lateral prefrontal cortex. This demonstrates that the very concept of brain the investigator himself most often implicitly presupposes may strongly impact what and how he measures brain function and which neuronal variables can and will be linked to psychotherapeutic change.

Another crucial input by the investigator is the behavioral task he employs in brain imaging to induce changes in neuronal activity. Brain imaging may be performed in resting state and or during an activation state with the latter requiring a specific behavioral task. The choice and selection of this behavioral task is of vital importance. Functionally, the behavioral task should be linked to the psychodynamic processes targeted by the psychotherapist in psychodynamic psychotherapy as well as to the client's symptoms, his subjective and behavioral complaints. Psychotherapeutic intervention may then be assumed to contribute to “normalize” abnormal reciprocal modulation in depression. This implies for the experimental designs that the behavioral task used in scanning should recruit those neural processes and mechanisms that supposedly mediate both the psychodynamic interventions and the client's symptoms. In addition to such psychodynamic and symptomatic requirements, the behavioral task needs to meet experimental demands. Such experimental demands include careful control conditions, behavioral and subjective measurement of the effects of the task itself, empirical linkage to the targeted neural processes and mechanisms, etc. The main problem here is to reconcile psychodynamic and symptomatic requirements with experimental demands. The unit of interest on both the psychodynamic and symptomatic level includes usually a mixture of several psychological, subjective, and behavioral variables which though on the experimental level need to be carefully controlled and spaced apart. Since the development of the behavioral task, the activation paradigm, is vital, we will discuss this issue in more detail in the next section.

### Translation problem

**Figure 2:**
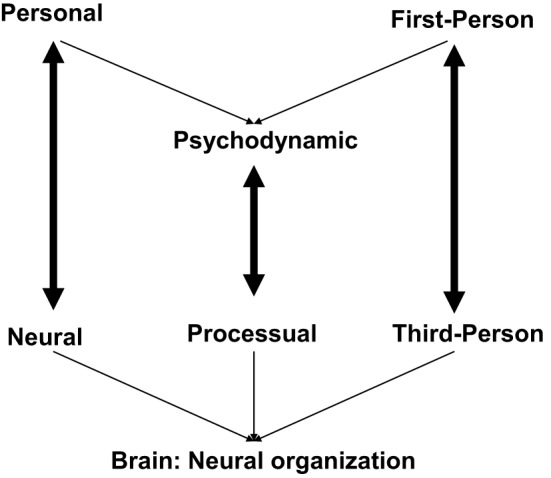
**Different levels and the translation problem**.

The above description of the design problem in its various facets reveals that different levels of investigation are involved. The translation problem deals with the methods and strategy how we can bridge the gap between the different levels. To simplify things, we want to discuss in the following four examples with each showing one pair of different levels. We will contrast personal and neuronal levels, psychodynamic and processual levels, and first- vs. third-person levels (see Figure 2).

#### Personal and neuronal levels: persons vs. brains

The psychotherapist and the client are individual subjects and must therefore be characterized as persons. The brain, in contrast, is not a person but rather an object. Though this seems obvious it has major implications in both conceptual and empirical regard. Let us consider first the conceptual implications. Bennett and Hacker (2003) warn not to confuse individual subjects with their brains because that means to neglect the basic difference between persons and objects; they speak of what they call mereological fallacy where the whole, i.e., the person, is confused with one of its parts, the brain. This means for instance that one cannot say that the brain thinks, feels, or acts since these attributes belong only to persons.

What is treated in psychodynamic psychotherapy is not the brain but the person. We may treat the person in a neurophysiologically-constrained way by considering neural processes and mechanisms in our psychotherapeutic interventions but this concerns only the neural processes that supposedly mediate the therapeutic outcome. Thus, to argue that we treat the client's brain rather than himself as person is not only to confuse person and brain but also to neglect the difference between neural processes/mechanisms and psychotherapeutic output. Neural processes and mechanisms concern the brain and may be regarded a necessary though not sufficient condition of psychotherapeutic output since other factors like interpersonal constellations, the cultural environment etc., have to be considered too.

The psychotherapeutic output, in contrast, concerns the level of the person that of course is somehow related to the brain but should at least conceptually not be identified with it. Hence, psychodynamic psychotherapy targets the person rather than the brain though its effects may, at least in part, be mediated neurally and thus by the brain. This implies that we should not aim to map the psychodynamic concepts in a one-to-one way with neural activity in particular brain regions or networks and thus to strive for what is described by the concept of “neural correlates.” This is so because that would mean to neglect the various other factors or variables that are implied and included in psychodynamic concepts as we saw in the specific case of psychotherapeutic intervention. Instead of the concept of neural correlates one may therefore want to preferably use the concept of “neural mechanisms” that, unlike the concept of neural correlates, does not presuppose one-to-one mapping between psychodynamic concepts and neural activity. As such neural mechanisms are supposed to underlie (rather than correlate with) psychodynamic concepts and thus psychotherapeutic interventions which leaves open conceptual and empirical space for including variables other than purely neural ones.

#### Personal and neuronal levels: generality vs. individuality

Another important empirical implication of the conceptual difference between persons and objects is the difference between individual and general levels. Persons concern individual subjects each with major idiosyncrasies both psychodynamically and neurally. The focus of psychodynamic psychotherapy is always on the individual, its specific subjective and personal contents as derived from its life history. Psychoanalysis gives us a conceptual framework to link these individual contents, as they are experienced from the inside of the experiencing person itself, to general structures of the psyche of persons, as they are observed from the outside by the observer. Neuroscience, in contrasts, concerns the brain as we can observe it from the outside; thereby however the individual person's specifics get lost because the experimental approach averages across different individual subjects. The difference between individuality and generality marks a principal difference between psychoanalysis and neuroscience which is nicely expressed by David Milrod (2002, pp. 22–23) in the following quote: “Neuroscientists strive to explain fundamental phenomena such as perception, consciousness, emotion, memory, etc., including the subtleties of their integration, and in this way build up an understanding of the basic functioning of the organism. In recent years they have included a study of the self as it integrates with consciousness, emotion, and awareness of the object … In short, they concern themselves with the universal and objective. Psychoanalysis, which has historically focused on the individual and has been more interested in ontology, has as its goal the understanding of protracted intrapsychic, interpersonal, and subjective functioning of the individual. It was in order to better understand that functioning that psychoanalysts had to deal with the self and its representation. In dealing with the self, the psychoanalyst is more likely to focus on the contents of the self and its representation, the state of stability or fragility it may possess … In other words, they focus on those elements that make each individual different from one another.”

How can we bridge the principal difference between the individual level of persons and the general level of brains? One way is to investigate only single cases and to focus on case studies (see for instance Solms and Lechevalier (2002) with regard to lesions patients as well as Overbeck et al., 2004; Rudolf et al., 2004; Lai et al., 2007; Lehto et al., 2008, for single case studies of psychodynamic psychotherapy and brain imaging). This however precludes a deeper insight into the neural processes and mechanisms that may eventually mediate psychotherapeutic output. What we need to develop are experimental designs and analyses that allow to take the individualized data as starting point and then to take and preserve these individual features as starting point for group analyses without averaging and generalizing them out into a group mean.

One may for instance imagine that the regions of interest in the individual subjects are taken as starting point for averaging and group analysis. The individual regions of interest may not only be determined and oriented on anatomical constraints but also psychodynamic constraints like the predominance of a certain psychodynamic mechanism. Another possibility is to group the individual subjects according to their subjective or psychodynamic profiles as revealed in empirical investigation of subjective experience. For instance, subjects with “high scores of introjection” may then be grouped together and compared with those showing “low scores of introjection.” One of the major methodological challenges in the future is thus to develop experimental designs and ways of analyses that allow to link individual and general features on the neural level in the same way Freud achieved it on the psychological level in such an ingenious way.

#### Personal and neuronal levels: content vs. organization

Another issue in this regard is the difference between neural contents and neural organization. Psychodynamic concepts may mirror the general organization of psychological activity which then may be manifest and realized in specific psychological contents of that individual person. This parallels to the neural level. We mentioned above that one may search for principles of neural integration rather than specific regions and networks. Specific regions and networks mirror what may be called neural contents and these are the targets in for instance the search for the neural correlates of consciousness (NCC) presupposing mere correlation and one-to-one mapping strategies. The principles of neural integration refer rather to the organization of neural activity and hence to what we call neural organization.

If one now searches for psychodynamic concepts in specific neural regions and networks, one may attempt to link structures of psychological organization with neural contents. This however may be doomed to fail because one then confuses the level of organization, as presupposed on the psychological level, with the level of contents, as implied by the neural level, with both remaining unable to match or correspond on a one-to-one basis. Instead, one may rather link psychodynamic concepts to the neural organization with both presupposing and implying analogous structures. This however remains rather speculative at this point (see Northoff, 2011, for a first attempt in this sense with regard to the self) since especially the principles and structures of neural organization, as distinguished from neural contents, remain to be explored.

#### Psychodynamic level vs. process level

One of the main issues is the translation of psychodynamic concepts into processes that then can be psychologically and neurally investigated. Consider again the example of introjection as a psychodynamic mechanism.

Introjection is considered a psychodynamic mechanism that, based on Mentzos (1995), can generally be determined as a form of appropriating and relating objects to the subjects in a personal way that is called internalization. Internalization includes three different mechanisms, identification, introjection and incorporation. These mechanisms of identification depend on different structural levels of the ego functions and of the personality. Incorporation describes that the subjects incorporates and integrates objects into itself so that the object becomes part of the subject itself with the former being indistinguishable from the latter. The subject may also introject the object.

What distinguishes introjection from identification and incorporation (cf. Meissner, 1978)? The separate reality of the object is acknowledged by the subject in introjection but the object relations are highly ambivalent including aggressive and narcissistic conflicts and feelings of anxiety, which are defended by projective mechanisms. In contrast, identifications depend on differentiated, continuous object relations and enable a selective internalization of partial aspects of the object. Ambivalent emotions may be tolerated and expressed. Incorporations, introjections, and identifications are important steps and components of the maturation process. Disturbances of the maturation process may lead—in a psychological developmental and psychoanalytical perspective—to the development of pathological defense mechanisms and the reactivation of early modes of internalization and object relationships (e.g., introjection in depression and Borderline, see Boeker et al., 2006).

Introjection allows the distinction between subject and object by the subject; however the price for acknowledging their difference consists in ambivalence with subsequent affects and anxieties. Metaphorically speaking, the object becomes strongly affectively colored by the subject while at the same time retaining its separate reality and reality for the subject. By means of affective involvement, the object is thus subjectified and related to the subject or, as one could say with Mentzos (1995), something objective(-object) is transformed into something subjective(-object): the parenthesis are included because the object becomes only colored by subjectivity while retaining its status as object whereas in projection, as the opposite of introjection, one would probably speak of objective-subject. The result of this process of introjection may be what is called an introject, the internal representation of an object. An object can be internalized and introjected and thus become an introject only if it has a special meaning and personal significance to the subject which usually is reflected in strong emotional involvement with the respective emotional feelings. If, for, instance, somebody has a rather close but ambivalent and therefore a strongly emotionally loaded relationship to her/his mother, the mother as object may become internalized and introjected to resolve the ambiguity in the relationship resulting in the mother being an introject for the subject. If, in contrast, the relationship to the mother is positive and free of ambiguity, it is possible to identify with the mother selectively as well as being separated from her.

This short description of introjection points out some cardinal psychological processes like relating objects to the person's self which has recently been described as self-related processing (Northoff et al., 2006). Moreover, it is clear that emotion processing is involved and closely linked to self-relatedness. Furthermore, the ability to relate to other people that involves empathy is crucial in introjection. At the same time however self-awareness is also involved since otherwise the introjecting person remains unable to distinguish itself as subject from the object, i.e., from other persons. These psychological processes that then could be regarded as starting point for developing a neuro-psychodynamic hypothesis of introjection. Accordingly, a translation from the level of a psychodynamic concept, i.e., introjection, to a process level, i.e., self-related and emotional processing etc., is needed to develop neuro-psychodynamic hypotheses and appropriate experimental designs. The psychological processes that may eventually be involved in psychodynamic concepts may then be used as guiding thread for where to look in the brain and what kind of principles of neural organization may be involved.

#### First-person level vs. third-person level

Systematic examination and evaluation of subjective experience must preserve its richness and complexity on the one hand, and objectively quantify its main characteristics on the other. Objectification and quantification of subjective first-person data allows for scientific investigation and consequently for establishing what can be called a “science of experience” (Gabbard, 2000). Based on a “science of experience,” a “science of psychodynamic processes” needs to be developed. The “science of psychodynamic processes” should place great emphasis on patients' mental life or inner experience in order to preserve the richness and complexity of subjective experience and clinical description. At the same time, these subjective features must be objectified to provide reliable and quantifiable data. This can be achieved by asking the subjects to complete rating scales. For example, visual analog scales (Weinryb et al., 1991a,b) with regard to personal identity or idiographic instruments like the Repertory Grid Test (Boeker et al., 2000) which enables the evaluation of idiosyncratic experiences and views by means of a semi-quantitative measurement, might be applied to let the subjects themselves evaluate their experiences. One might also apply structured interviews with valid and reliable instruments for evaluation of the subjects' relevant psychodynamic features by an experienced investigator. General instruments include, for example, the Karolinska scale that assesses different psychodynamically-relevant dimensions of a person's structure (Weinryb et al., 1991a,b). Another instrument is the Operationalized Pychodynamic Diagnosis (OPD-2; OPD-Task Force, 2008) which examines three psychodynamically relevant axes interpersonal relations, conflict and psychic structure, an axis on the experience of the illness and prerequisites for treatment and one descriptive axis (psychic and psychosomatic disturbances according to ICD-10 and DSM-IV).

One of the main methodological challenges in investigating the neuronal processes underlying mechanisms is to link these first-person data about psychodynamic processes to third-person observation of neural states. Being based upon subjective experience, psychoanalysis relies on first-person data or more precisely on data obtained by introspection that presupposed what may be called Second-Person Perspective (which in the following we will subsume under the concept of First-Person Perspective). This contrasts with neuroscience which requires third-person observation of neuronal states. Due to the neglect of first-person subjective experience, neuronal states as third-person data can be quantified and objectified. This, in contrast, remains impossible in the case of first-person data which are rather qualitative and subjective. If, however, the neuronal processes of mechanisms are to be investigated, subjective experience and neuronal states (i.e., first- and third-person data) have to be linked to each other in a systematic way. For this purpose, we have created an appropriate methodological strategy, First-Person Neuroscience, which aims at systematically linking first- and third-person data (see Northoff, 2007) that also conceptualizes many investigations in current brain imaging that correlate subjective experiential variables (as for instance in visual analogs scales) with neural measures of brain function (see for instance Grimm et al., 2009).

We define “First-Person Neuroscience” as a methodological strategy to systematically link first-person subjective experience to third-person observation of neuronal states. The development of such methods distinguishes First-Person Neuroscience from neuroscience as it is commonly practiced which most often relies on third-person observation of neuronal states more or less independently of subjective experience. The main challenge in establishing First-Person Neuroscience consists in linking the individual contents of subjective experience to neuronal states. How can we link subjective experience to neuronal states?

Linkage between subjective experience and neuronal states requires two steps: first, subjective experience needs to be evaluated systematically including objectification and quantification of subjective data. Such “science of experience” is a necessary precondition for any linkage between subjective experience and neuronal states. Second, the systematically objectified and quantified subjective data then enable the linkage to analogous data about neuronal states. For this, special methodological strategies need to be developed—this is the core of what we call “First-Person Neuroscience.” The above described discussion of how to translate the psychodynamic concept of introjection, that experimentally is accounted for by first-person data, into a behavioral task as activation paradigm, that yields third-person data about the brain, can be regarded as example of how to link first- and third-person perspectives and may therefore be regarded an instance of First-Person Neuroscience.

## Individualized paradigms in neuro-imaging: state of the art

### Existing approaches

Over the past years, a number of studies have employed neuro-imaging methods in psychoanalytically oriented research. The importance of individualized experiments in studying psychotherapeutic changes, for instance, has been stressed by Kessler et al. (2011b, 2012). Though neurobiological changes in some (single) cases undergoing psychodynamic psychotherapy have been reported, mostly using Single-photon Emission Computed Tomography (SPECT) (Viinamäki et al., 1998; Saarinen et al., 2005; Lai et al., 2007; Lehto et al., 2008) with few using functional Magnetic Resonance Imaging (fMRI) in studies of obsessive compulsive disorder, panic disorder, and somatoform disorder (Overbeck et al., 2004; Beutel et al., 2010; deGreck et al., 2011). To date, studies examining the functional neuroanatomy of psychotherapy in depressed patients involved IPT or CBT (Roffman et al., 2005; Linden, 2006). Buchheim et al. (2012) were the first to conduct an fMRI study with depressed patients treated with psychodynamic psychotherapy using two fMRI paradigms (Kessler et al., 2011a, 2012; Taubner et al., 2012). The first paradigm was based on clinical material drawn from OPD-2 interviews. Kessler et al. (2011a, 2012) confronted patients with themes of their maladaptive interpersonal relationship patterns, presenting them sentences in the scanner which were derived from an OPD interview. They individually selected four sentences for each person representing a core dysfunctional relationship theme. During the control condition (traffic) patients recalled a stressful traffic situation they had experienced inducing negative emotions and recalling autobiographical memories. Conditions were separated by a “relaxation” condition. The second paradigm, described by Taubner et al. (2012), used attachment-related pictures from the Adult Attachment Projective Picture System (AAP). They aimed at eliciting mental engagement with attachment-related experiences such as loss, illness, danger, and separation. During an AAP interview patients described the scene in the picture including what characters were thinking and what could happen next. From this interview three sentences representing the attachment pattern of each patient were extracted. As control condition participants were shown the AAP pictures and were invited to only describe the environment depicted.

Methodological shortcomings in these fMRI paradigms can be discussed such as the elevated cognitive demand implied by a reading task, unsatisfying control conditions and difficulty concerning the selection of stimuli. These methodological concerns are expression of the complex endeavor that the investigation of neural mechanisms of subjective experience implies (Logothetis, 2008). Developing valid experimental designs taking into account the very individual dimension of experience is an arduous methodological challenge, as it has been illustrated here before. In the following section, our attempt to create a new experimental design for the investigation of neural mechanisms in depressed patients during psychodynamic psychotherapy will be depicted.

Coherent with the above-described methodological strategy named “First-Person-Neuroscience” the experimental design aims at systematically linking subjective experience to the observation of neural states. This necessitates the objective evaluation of subjective experience in a first step. In a second step, the so created data has to be linked to observations on the neural level.

To begin with, we will outline our choices of how to evaluate subjective experience, giving a brief description of the axis “interpersonal relations” from the OPD-2 (OPD-Task Force, 2008) as well as the Maladaptive Interpersonal Patterns Q-Sort (MIPQS; Zimmermann et al., accepted). We will then describe the development of the “Interpersonal Relations Picture Set” (IRPS) and a new neuro-imaging experiment based on the IRPS.

### The OPD-2 axis “interpersonal relations” and the maladaptive interpersonal patterns Q-Sort (MIPQS)

An adapted instrument to attempt an objective evaluation of subjective experience is the OPD-2 (OPD-Task Force, 2008). It operationalizes psychodynamic dimensions in different diagnostic axes. Regarding depressive disorders, the axis “interpersonal relations” is particularly relevant, as depressive disorders go along with various impairments in interpersonal and social functioning. For example, depressive patients tend to have deficits in emotional expression and emotion recognition in others. They also tend to have difficulties with affective modulation in basic interpersonal communication of emotions and feelings. Broadly speaking, psychodynamic psychotherapy sets a specific focus on these affective processes using phenomena of transference and countertransference. The axis “interpersonal relations” of the OPD-2 offers a classificatory system describing different patterns in interpersonal behavior. This diagnostic axis enables clinicians to assess and precisely describe specific maladaptive interpersonal patterns in patients with depression.

Recently, the Maladaptive Interpersonal Patterns Q-Sort (MIPQS), a self-report version of the OPD-2 axis “interpersonal relations,” has been developed (Zimmermann et al., accepted). Using a card sorting procedure, the MIPQS allows the establishment of a subjective and hierarchized profile of typical interpersonal behavior. One of its substantial advantages compared to the OPD interpersonal relation axis is the facility in its use. The MIPQS exists in two versions. The MIPQS-A is a self-evaluation of the participant concerning typical interpersonal behavior. In contrast, the MIPQS-B enables the clinician to evaluate the participant's interpersonal behavioral patterns from his point of view and based on the interview situation. We will concentrate here on the MIPQS-A. The card sorting procedure of the MIPQS-A (named MIPQS in the following) comprises two steps. In a first step, the participant rates the relevance of behavioral patterns (described by 32 items) in his own behavior toward significant others. In a second step, the participant rates the relevance of described behavioral patterns (equally 32 items) in the behavior of others toward himself. The description of patterns in interpersonal behavior was adopted from rating items of the OPD interpersonal relation axis. These items are theoretically close to interpersonal circumflex models such as used in interpersonal psychology (e.g., the SASB/Structural Assessment of Social Behavior model, Benjamin et al., 2006). The SASB model describes different qualities of interpersonal behavior by means of two orthogonal and bipolar dimensions: control (dominant vs. submissive) and affiliation (friendly vs. hostile). The MIPQS items have been empirically tested and can be located in the circumplex model comprising these two dimensions. Every one of the 32 MIPQS items consists of two easy to understand descriptions of a specific interpersonal behavior pattern like “I tend to ignore others or give them the cold shoulder” and respectively “Others tend to ignore me or give me a cold shoulder.” They are printed on separate cards. The sorting procedure includes the depositing of the 32 cards on finally nine columns ranging from “most typical” to “most untypical” with “unimportant” as the fifth column. Furthermore, the instrument offers a sequential ranking of all items from 1 (most untypical) to 32 (most typical).

Based on the items of the MIPQS we have developed graphic illustrations forming the so-called “Interpersonal Relations Picture Set” (IRPS), which we plan to use in fMRI experiments. In the following the different steps of its development will be described.

## The interpersonal relations picture set (IRPS)

**Figure 3:**
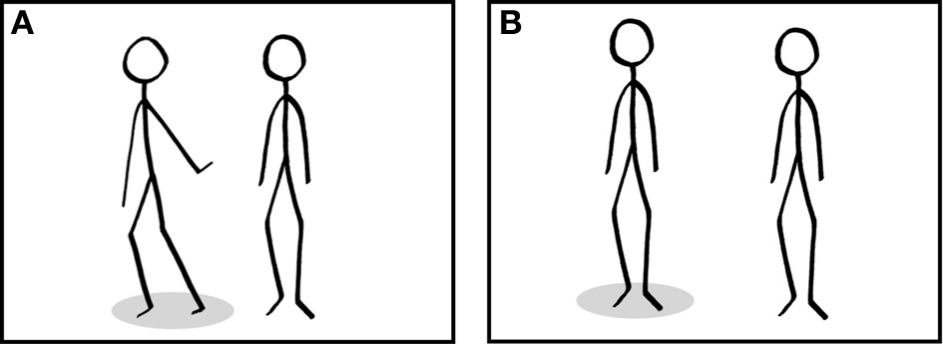
**Example from the IRPS representing the item “I tend to ignore others or give them the cold shoulder” (A) and an example of a picture from the control condition (B) used in the fMRI-experiment.** Please note that style and number of the stick figures and the basic characteristics of the pictures are matched across conditions. Subjects are asked to take the interpersonal perspective of the figure indicated by the gray marker.

The MIPQS provides 32 items describing patterns of interpersonal behavior. The IRPS comprises pictures illustrating interpersonal behavior patterns. The pictures were developed step-by-step. At first, there had been an attempt to illustrate the interpersonal situations by means of multicolored symbols. However, this approach was abandoned because of the high level of abstraction of these symbols. Consecutively, a collection of pictures illustrating the different situations by means of stick figures was composed (see Figure 3A). The pen-drawings were scanned for further processing in a widely used image editor (Seashore^©^). Each picture shows two or more black stick figures on white background. The figures vary in size, but do not show any gender specification, facial expression, clothes, or other specific characteristics. Some figures, e.g., the figure taking a neutral body position, occur repeatedly in different pictures. Different interpersonal situations are expressed only in the specific posture of figures as well as their positions toward each other.

### Validation of the pictures

Concerning the testing of these pictures, ethical approval, and permission was obtained for all procedures described below from the Ethics Committee at the University of Zurich. A complete and detailed description of the study was provided to participants and patients and they gave a written informed consent concerning their participation.

In order to confirm the relation of every picture to the assigned item of the MIPQS we performed an online survey. It was sent to a mailing list of the university. The survey included an introduction, an instruction concerning the questionnaire and a request of age, gender, educational level, and history of interpersonal relationships. Then the ISPS pictures were presented in randomized order. Participants were asked to judge the pictures in two conditions. Firstly, in the “discrimination task,” pictures were presented combined with the matching description as well as four randomly selected descriptions out of the remaining 31 MIPQS items. Furthermore, a response that none of the offered descriptions fitted the picture was added. This form of questioning was designed as multiple choice task and only one answer was allowed. Participants were asked to distinguish the matching description from the offered choice. Secondly, in the “relevance task,” participants were asked to rate the level of relevance of each picture. A nine-point Likert scale was combined with the picture and its matching description for this task. The sample of the online survey was randomly split into two groups to avoid a repetition effect. Hence, no item was presented twice in the survey. Statistical analysis was conducted using the SPSS® software package (IBM). The data for all variables collected were subjected to descriptive statistics according to the respective scale level. In order to assure direct interpretability of results of both tasks, the data analysis was limited to descriptive statistics. This included indicating the proportion of correct responses for multiple choice tasks in percentage and the median for nine-point Likert scales.

In a further step, we tested the correspondence between the IRPS and the MIPQS when participants performed them separately. A number of patients suffering from Major Depressive Disorder (MDD) were recruited from the Department for Psychiatry, Psychotherapy, and Psychosomatics of the University Hospital of Psychiatry Zurich. Patients with neurological or other physical illnesses, disorders of personality, alcohol- or substance abuse were excluded. Diagnosis was made according to DSM IV (American Psychiatric Association, 1994). Clinical symptoms were assessed with the BDI II (Beck et al., 1996) and the Hamilton Depression Scale (Hamilton, 1960). After execution of the MIPQS, patients were asked to perform the relevance task (as described above) using the experimental control software Presentation® (Neurobehavioral Systems). Ratings for each item were related to the number of the column chosen by the patient in the MIPQS using Spearman rank correlations.

### Course of the experiment

The fMRI experiment is embedded in a large-scale psychotherapy outcome study. We investigate changes in depressed patients during 1 year of psychodynamic psychotherapy concerning psychodynamic, behavioral and neuronal parameters. Different instruments are employed for the evaluation of these changes, such as the OPD interview, fMRI examination and a series of questionnaires: MIPQS (Maladaptive Interpersonal Patterns Q-Sort; Zimmermann et al., accepted), OPD-SF (“OPD Struktur-Fragebogen,” [OPD Structure Questionnaire]; Ehrenthal et al., 2012), BDI (Beck et al., 1996), BAI (Beck Anxiety Inventory), BHI (Beck Helplessness Inventory), FKBS (“Fragebogen zu Konfliktbewältigungsstrategien,” [Questionnaire on Coping with Conflicts]; Hentschel et al., 1998), IIP-D (Inventar zur Erfassung Interpersonaler Probleme [Inventory of Interpersonal Problems] Horowitz et al., 2000), HCSC (“Heidelberger Umstrukturierungsskala,” [Heidelberg Structural Change Scale]). A specific focus is set on changes in interpersonal behavioral patterns such as evaluated by the MIPQS. The IRPS will be employed during the fMRI examinations. The procedure for the fMRI experiment will roughly be described here after.

In a first step, participants rate the MIPQS and the IRPS. Six pictures representing the most typical interpersonal situations for each participant are selected. Participants are then invited to develop a personal narrative for every one of the six target pictures. Ideally, this determines the individual meaning of each one of these stimuli. After, participants proceed to fMRI examinations. Instructions include a structured description of the task. Before proceeding to the fMRI examination, participants perform a trial run. Stimuli are presented in a block-design in randomized order. Four experimental conditions are used in the scanning procedure. In a first condition (“typical”) the six target pictures are presented. A second condition (“untypical”) consists of pictures rated beforehand by the participants as being not typically representative for their interpersonal behavior. The control condition (“neutral”) includes pictures showing a number from two to four stick figures in a frontal, neutral position (see Figure 3B). Finally, a resting condition (“rest”) showing a black fix-cross on white background is included. In order to evaluate subjective experience during the “Typical” and “Untypical” condition, a nine-point Likert scale is presented after every picture during the scanning procedure. Participants are asked to rate the level of personal involvement experienced while watching the pictures. In the “neutral” condition subjects are asked to report the number of stick figures presented in every picture. Stimuli are presented using Presentation® and all feedbacks are given using a trackball response pad (Current Designs®). The experimental design was optimized for further analysis of effective connectivity.

### Discussion

We have illustrated our attempt to develop an individualized neuro-imaging paradigm. As mentioned before, the choice and selection of the behavioral task employed during fMRI examinations is of vital importance. We have chosen to base the behavioral task on a validated instrument (MIPQS) describing a central dimension in psychodynamic psychotherapy: changes in interpersonal behavior and associated feelings. We hence try to isolate a specific mechanism relevant in the psychodynamic treatment of depression and operationalize it in an fMRI experiment. The experiment incorporates a number of specific principles that are, in our view, of great importance for this type of experimental design.

First, focus is set on the very individual dimension of experience as well as their emotional implications. This is reflected in the individual choice and subjective determination of meaning of stimuli (IRPS pictures) used during neuro-imaging.

Second, the association of the picture to autobiographical experience should strengthen the affective reaction of the participant when the IRPS pictures are presented in the scanner. During the scanner procedure, participants rate their subjective emotional arousal induced by the IRSP pictures. Having a subjective rating of this kind enhances the validity of the experiment.

Third, the use of visual stimuli in form of pictures may reduce the cognitive demand on participants during fMRI examinations compared to tasks involving stimuli using words or sentences.

Fourth, the fMRI experiment comprises a valid control condition. The control condition consists in presentation of (a) IRPS pictures that the patient rated as non-relevant for himself; (b) pictures showing stick figures in a neutral position.

Results of fMRI exams will be linked to results from other diagnostic instruments such as the OPD and a series of others questionnaires. By the choice of these instruments, we tempt to take into account the complexity of subjective experience.

There are several factors that have been pointed out earlier to be relevant for the design of neuro-imaging paradigms in psychodynamic research that are not taken into account in our paradigm. This includes factors resumed under the “design problem” as well as those evoked concerning the “translational problem.” For example, we have not considered the psychotherapist or the therapeutic relationship as “input” in depth. Our design includes one questionnaire possibly giving a hint on the matching of therapist and patient (IIP-D) but it does not include personality or attachment style ratings for the therapist. Ideally, this should also be taken into account. To give another example, we also need to carefully consider our hypothesis concerning neural activation during fMRI exams and take into account considerations illustrated earlier with reference to “the investigator as input.”

Our experimental paradigm does not aspire to satisfy all of the requirements that experimental designs in psychodynamic psychotherapy research using neuro-imaging should ideally fulfill and which were described earlier in this article. In this vast and complex research domain, the development of adequate and valid experimental designs stays a defiant methodological challenge. Our experimental paradigm represents a further step into this direction. It aspires to create an experimental design that does reflect—even though in a limited way—the complexity of subjective experience.

## Conclusion

We discussed the methodological problems in designing a brain imaging study to measure neural effects of psychodynamic psychotherapy. Two main problems, the design problem and the translation problem, were encountered. The design problem points to the many inputs including the psychotherapist himself, the client, and the investigator, which each by itself may need to be included as distinct experimental variables in the study design. The translation problem refers to the different levels involved in such project such as the personal vs. the neuronal level, the psychodynamic vs. the process level, and the First-Person level vs. the Third-Person level. Thereby the personal vs. the neuronal level is of particular interest in that it includes conceptually and empirically relevant distinctions like persons vs. brains, generality vs. individuality, and organization vs. content.

Taken together, this demonstrates that brain imaging studies of the neural effects of psychodynamic psychotherapy are confronted with a rather high degree of complexity raising various conceptual, empirical, and experimental problems. The discussion of these problems shall not discourage future investigators; instead it shall provide them with some suggestions for guidance through the jungle of complexity. Though any such investigation requires multi-professional efforts and emphatic collaboration, we are sure the merits are highly rewarding. The complexity of investigating the neural effects of psychodynamic psychotherapy mirrors in an almost paradigmatic way the complexity of our brain so that neuro-psychodynamic findings entail insight and a better understanding of the general principles of neural organization and our brain's very human nature. The answer to this question is two-fold: on one hand we do think that by revealing the neural mechanisms underlying psychotherapeutic processes, we may be able to develop more specific protocols of psychotherapy in orientation to the respective neural functions associated with the respective region. For instance, taking a rather simplistic example, if the level of neural activity in the amygdala may be involved in psychotherapeutic processes and even be predictive of psychotherapeutic outcome, it may be an additional indicator that the involvement of emotions may have been crucial in psychotherapeutic success. This may be the case even if the therapy was not emotion-focused but rather oriented in psychodynamic mechanisms. Such results may then be considered as evidence for the central involvement of emotional processes in the psychodynamic processes which may then lead to further refinement and specification of psychotherapeutic protocols. This raises not only the question for the linkage between emotions and psychodynamic processes but also how we, for example, can address emotions more explicitly in relation to defense mechanisms in psychotherapy. Such orientation on neural functions may then lead to the development of neurally-based psychodynamic psychotherapy in the future and may therefore be empirically, i.e., neurally, more plausible, and compatible with respect to the brain and its mode of function than the current purely clinically- and observationally-based approaches.

On the other hand, revealing the neural mechanisms underlying psychotherapeutic processes may also contribute in the reverse direction, by giving us a better understanding of the psychological, i.e., psychodynamic, mechanisms associated with certain patterns of neural activity across different region. Hence, it is not only that psychodynamic psychotherapy may benefit from brain imaging but also the other way in that the latter may also be complemented by the latter.

In summary, we thus assume bilateral exchange and contribution between psychodynamic psychotherapy and brain imaging. This may ultimately, as we hope, lead to the development of diagnostic and therapeutic predictive markers with especially the latter predicting what subjects may benefit from what kind of psychotherapy in general, and the kind of focus in psychodynamic psychotherapy in particular.

### Conflict of interest statement

The authors declare that the research was conducted in the absence of any commercial or financial relationships that could be construed as a potential conflict of interest.
